# The Effects of a Real-Time Visual Kinetic Feedback Intervention on Shock Attenuation of the Equestrian Rider's Trunk: A Pilot Study

**DOI:** 10.3389/fspor.2022.899379

**Published:** 2022-06-22

**Authors:** Marc Elmeua González, Nejc Šarabon

**Affiliations:** ^1^Faculty of Health Sciences, University of Primorska, Izola, Slovenia; ^2^Laboratory for Motor Control and Motor Behaviour, S2P, Science to Practice, Ltd., Ljubljana, Slovenia; ^3^Human Health Department, InnoRenewCoE, Izola, Slovenia

**Keywords:** equitation sciences, motor learning, augmented feedback, stirrup forces, rein forces

## Abstract

Augmented feedback (provided by an external source) has been commonly used by practitioners who are introducing or re-educating movement patterns as a valuable tool of instruction. This study aimed to evaluate the effects of real-time visual kinetic feedback on a horse-riding coaching session. Sixteen riders volunteered to take part in this study. They performed a pre-intervention trial, a 20-min coaching intervention, and a post-intervention trial. The participants randomly received a coaching + feedback intervention or a coaching-only intervention. Forces at the bit and stirrups were recorded at trot and canter. Thirteen inertial measuring units were fitted to the horse's forelimbs and poll, to the stirrups, cantle of the saddle, distal part of the bridles, 1st sacrum vertebrae of the rider (S1), 7th cervical vertebrae of the rider (C7), wrists of the rider, and helmet. The shock attenuation (SA) between helmet:saddle and between C7:S1 and absolute force output were calculated. Changes in SA and force output were compared between groups by two-way repeated measures ANOVA (group^*^time) both at trot and canter. Statistical significance was set at *p* < 0.05. SA was significantly lower in both groups and conditions after the intervention. C7:S1 SA was significantly lower in the feedback + coaching group at canter and trot, and helmet:saddle SA was significantly lower in the feedback + coaching group at trot than in the coaching group. A significant increase in force was observed in all the groups on the stirrups at trot and canter, but no significant changes were observed on rein forces. Implementing sports wearables that provide such type of information might be of remarkable benefit for the rider's development and performance.

## Introduction

Augmented feedback (i.e., information on performance provided by an external source) has been proposed as a key tool for coaching new skills, and it can be divided into either knowledge of performance feedback (KP) or knowledge of result feedback (KR) (Lauber and Keller, 2014) depending on whether the information received by the learner is related to the quality of the movement pattern (KP) or the success in achieving a spatiotemporal goal (KR). The sound of a horse hoof against a jumping fence would be a KR cue suggesting that the equestrian dyad has not successfully cleared a jump, whereas a coach pointing out an unbalanced seat of the rider that led to a fallen fence would be a KP cue. It is unclear if one strategy is better than the other or whether they work better in conjunction than alone (Zubiaur González et al., 1999; Hinder et al., 2008, 2009; Drews et al., 2021). Nonetheless, there seems to be a trend that favors the use of KP for complex tasks and tasks developed in unstable environments such as horse riding (Hinder et al., 2008, 2009; Bishop et al., 2018; Ahulló et al., 2019).

Augmented feedback can also be divided into the nature of the information being transmitted. Lauber and Keller (2014) proposed three types of augmented feedback: (i) kinetic feedback, which refers to forces and torque outputs of the learner, (ii) kinematic feedback, which is associated with the learner's movement-related aspects, and (iii) biofeedback, which is linked to physiological responses. Furthermore, feedback strategies can be classified into concurrent (real-time) and terminal (after the task has been completed) (Sigrist et al., 2013).

Augmented feedback can be mainly visual, auditory, haptic, or multimodal. Tasks differing on their nature and complexity require different types of feedback. In this regard, concurrent visual feedback has been more successfully applied in complex tasks (Sigrist et al., 2013). Crowell et al. (2010) suggested that real-time visual feedback had the potential to reduce the impact loading at the tibia in trained runners. Hence, similar results could be expected from horse riders trying to attenuate impact loadings at the spine.

The goal of this study is to test the effects of a coaching intervention combined with real-time, visual, kinetic, and KP augmented feedback on the attenuation of shockwaves on the rider's spine and the force output on stirrups and reins, both at posting trot and collected canter. We hypothesized that implementing the aforementioned form of feedback will lead to lower shock attenuation values associated with increased stirrup forces.

## Methods

### Participants

Eleven recreational horse riders (26 ± 8 years, training 2–4 h/week, and 12 ± 5 years of experience) volunteered to participate in the study. They were informed of the study's procedure and signed informed consent. The National Medical Ethics Committee (Ministry of Health, Ljubljana) gave full approval to the project according to the Declaration of Helsinki (approval number: 0120-346/2018/3). One horse (9 years) was used.

### Experimental Protocol

All measures were taken at posting trot and collected canter.

A repeated measures, single-visit study design was used. All measures took part on a standard indoor arena 20 × 40 m and lasted <2 h. The horse rested > 24 h between trials. After obtaining the informed consent of the participants, thirteen inertial measuring units (IMUs) (DelsysTrigno, Natick, MA, United States; 2017) were placed on the rider, saddle, bridles, and horse (refer to Inertial measures for more information). After a 12-min standardized warm-up of the horse and the rider (2 min at walk, trot, and canter, on both leads), the participants performed a baseline trial (120 trot strides and 120 canter strides on both the right and left leads). Then, the participants randomly went through either a 15-min coaching intervention or a coaching + visual feedback intervention. Finally, the participants were required to perform a post-intervention trial (60 trot strides and 60 canter strides on both the right and left leads). None of the participants had ridden the horse before, but they had the opportunity to get familiar with it during the warm-up.

### Interventions Design

One group (control) was instructed by an accredited coach with concurrent KP verbal feedback. Only the following cues were allowed: *step hard on the stirrups, lower/rise your wrist, pull back your ankle, place your toe under your hip, lean forward/backward/left/right, make a straight line with the rein from your hands to the bit, apply more/less pressure, look to the front*, and *straighten your back*.

**Figure 1:**
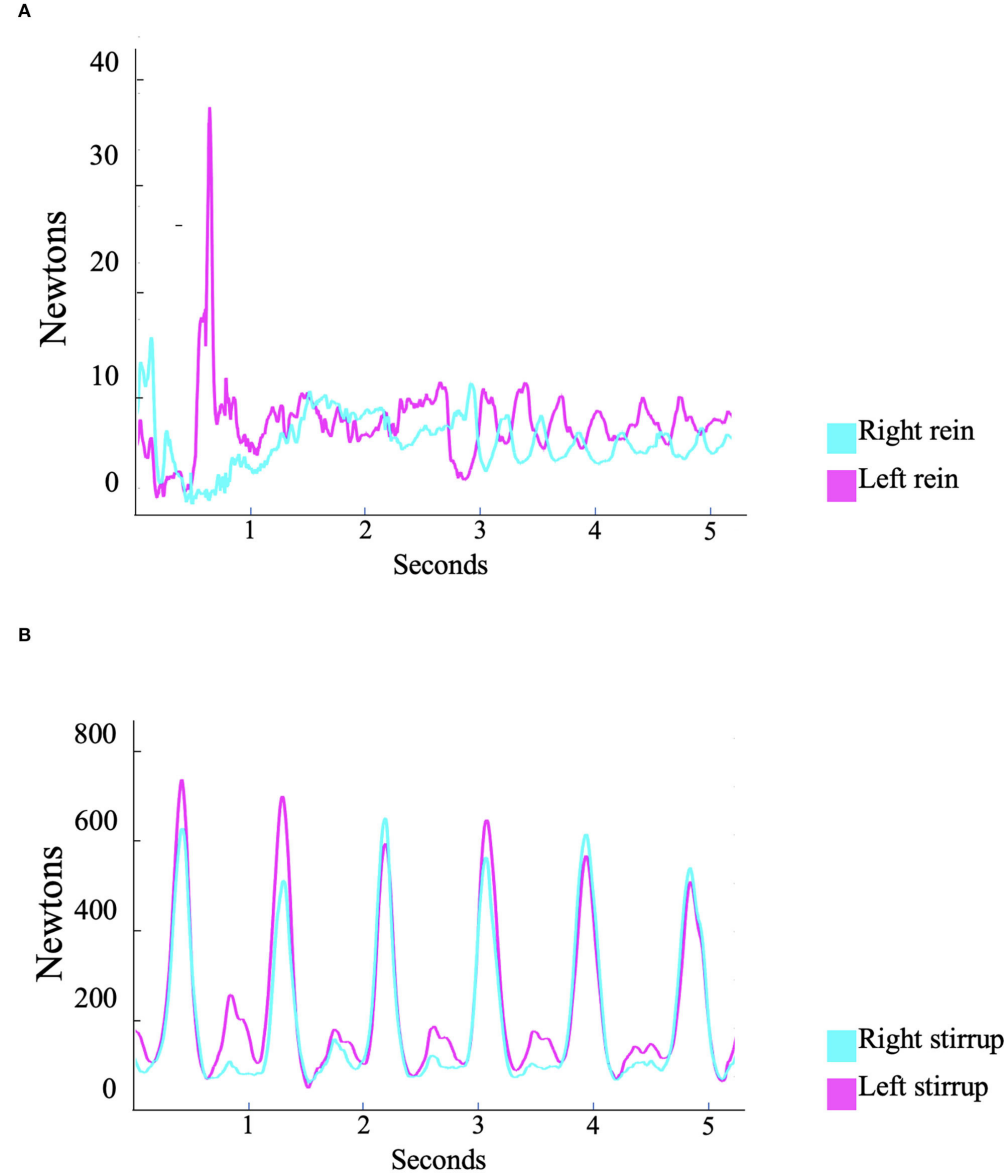
Real-time feedback on the tension exerted at the **(A)** reins and **(B)** stirrups of a representative subject while being coached to achieve steady and balanced forces. The feedback included rein and stirrup forces displayed as a time-series line chart. With this implementation, the rider was able to continuously visualize how hard or soft he or she was in pushing down the stirrups or pulling on the reins.

Another group (experimental) was instructed by the same coach with the same cueing instructions and was provided with real-time feedback on a tablet screen that was mounted on the pommel of the saddle and displayed rein and stirrup forces (Figure 1).

The coach was requested to get the riders to improve their balance and lightness on the bit, and increase contact on the stirrups.

### Horse Setup

An all-purpose saddle was used. Saddle tilt and stirrup length were set neutral as described in (Elmeua González and, 2021). The bridle was mounted with a simple snaffle bit.

### Force Measures

Forces between the reins and the rings of the snaffle bit and between the stirrups and the stirrup leathers were registered with a uniaxial tension load cell (FL 5–25 and FL 35–200 kg respectively; Forsentek Co., Ltd., China). Each sensor was cabled to a single-channel amplifier (INSAmp; ISOTEL, Slovenia) and then routed to a data logger through a multichannel acquisition card (NI USB-6218; National Instruments, United States). The data logger and acquisition card were tightly mounted on the rider's back. Forces were recorded with custom-made software (S2P d.o.o., Slovenia) developed with LabView 2015 (National Instruments, United States) at a sampling rate of 1,000 Hz. A 3-point calibration was conducted for the sensors, with known weights of 10, 15, and 20 kg for the snaffle sensors and 40, 100, and 150 kg for the stirrup sensors.

### Inertial Measures

**Figure 2:**
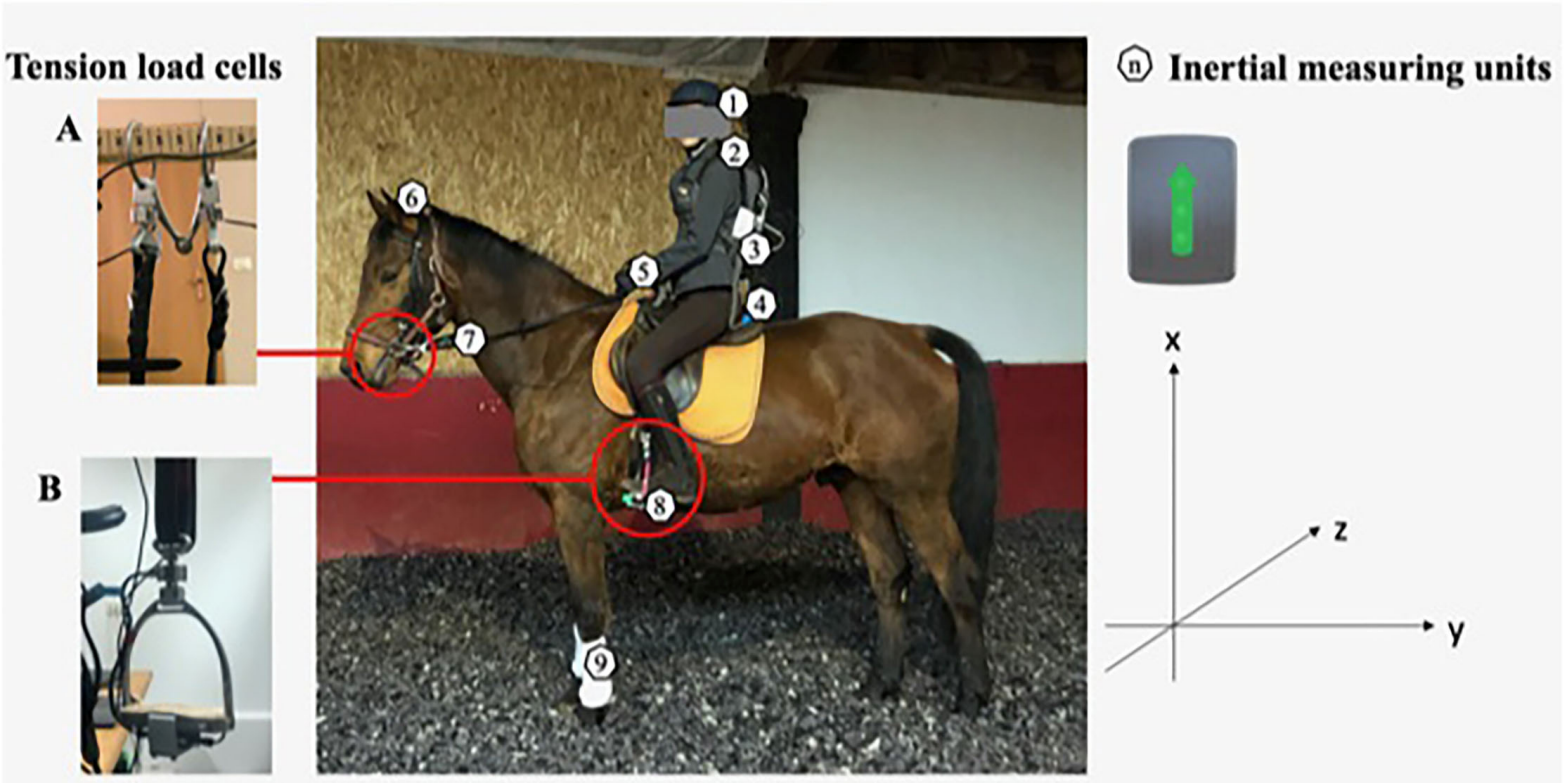
Placement and orientation of the tension load cells of the **(A)** bridles and **(B)** stirrups and the inertial measuring units (IMUs). (1) Helmet, (2) C7, (3) S1, (4) cantle of the saddle, (5) wrists, (6) horse poll, (7) distal part of the rein, (8) base of the stirrup, and (9) forelimb of the horse. Forces at the junction between the reins and the rings of the snaffle bit, and the junction between the stirrups and the stirrup leathers were registered.

Thirteen wireless IMUs (Delsys Trigno; Delsys, Natick, MA, United States) were placed on the horserider dyad (Figure 2). Anatomical locations were determined by manual palpation assuming the most prominent vertebrae on the cervical region was the 7^th^ cervical vertebrae (C7), and then counting down from the spine to the anterior sacral promontory (S1). The sensors were mounted directly with two-sided adhesive tape and were secured with kinesiology (KT^TM^) tape to the rider's skin in order to reduce their vibration and oscillation. Each IMU weighed 25 g and had a 27 × 46 × 13 mm body. The raw signal was amplified (gain: 2,000) and recorded at a sampling rate of 150 Hz *via* the built-in amplifier.

### Data Analysis

All signals were processed using MATLAB R2020b (Natick, MA, United States). Acceleration and force values were averaged across each condition. Acceleration and deceleration phases were eliminated by visually inspecting the IMU data. Data processing steps are shown in Figure 4.

Shock attenuation (SA) was calculated from accelerations at helmet:saddle -as a measure of attenuation between the horse-rider complex- and C7:S1 -as a measure of attenuation within the rider's back-. Acceleration data were filtered using a second-order, zero-phase digital Butterworth filter with a high-pass cutoff at 10 Hz and a low-pass cutoff at 60 Hz (Castillo and Lieberman, 2018). Such attenuations were measured using a transfer function given in decibels (dB) as:


$$\begin{array}{l} \text{SA}=\;10\text{Lo}{\text{g}}_{10}\;\left(\text{ACChigh}/\text{ACClow}\right) \end{array}$$

ACChigh and ACClow represent, respectively, the power spectral densities (PSD) of the accelerations recorded with the highest and lowest positioned accelerometers with regard to the vertical plane. PSDs were calculated from detrended accelerometer data by fast Fourier transformation from 0 to the Nyquist frequencies, found using a rectangular window. Details can be found in (Castillo and Lieberman, 2018).

Forces below the 5^th^ percentile and above the 95^th^ percentile were eliminated, as they were assumed to be created by extraneous events such as the horse stumbling (Eisersiö et al., 2015). Then, each condition bout was averaged.

### Statistical Analyses

All statistical analyses were performed with SPSS 23 (IBM Corp., Armonk, NY, United States), MATLAB R2020b (MathWorks, Natick, MA, United States), and MS Excel 16.24 (Microsoft, Redmond, WA, United States). Change in each SA ratio and force output of each sensor were individually compared between groups by two-way repeated measures ANOVA (RM-ANOVA) (group^*^time) both at trot and canter. Values are presented as mean ± SD. All the analyses were performed at an alpha level of 0.05.

## Results

### Shock Attenuation

**Figure 3:**
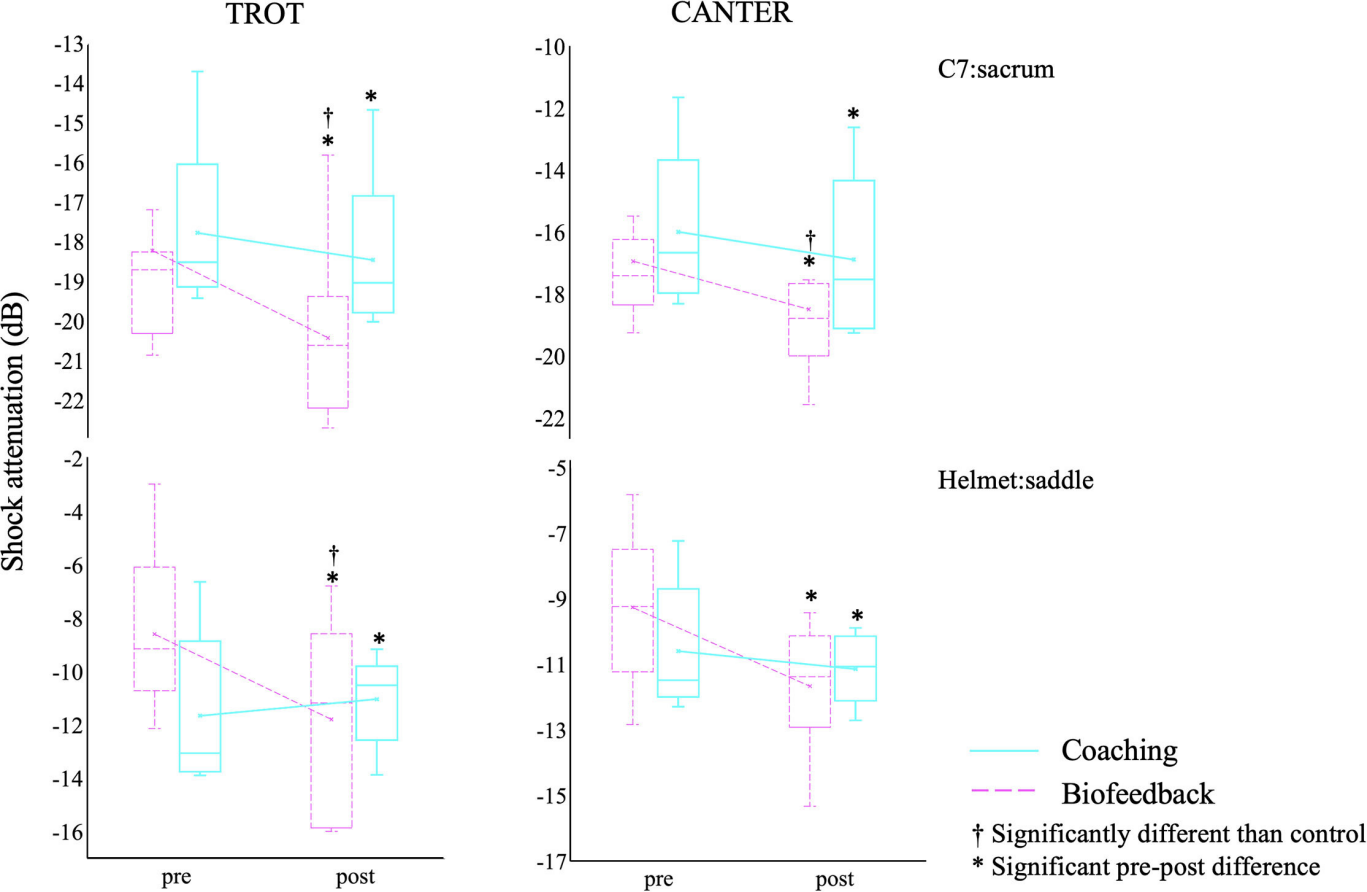
Boxplots of C7: sacrum and helmet: saddle shock attenuation waves before and after the coaching and feedback interventions at trot and canter. *Significant time interaction; † significant group interaction.

The two-way RM-ANOVA revealed a statistically significant interaction between group and time on C7:sacrum SA waves at canter [F(1, 15) = 4.598, *p* < 0.05] and trot [F(1, 15) = 4.395, *p* < 0.05], and on helmet:saddle SA waves at trot [F(1, 15) = 15.539, *p* < 0.001] (Figure 3). The simple main effects analysis revealed lower C7:sacrum SA waves in the feedback group at canter and trot (*p* < 0.05), and lower helmet:saddle SA waves in the feedback group at trot (*p* < 0.001).

**Figure 4:**
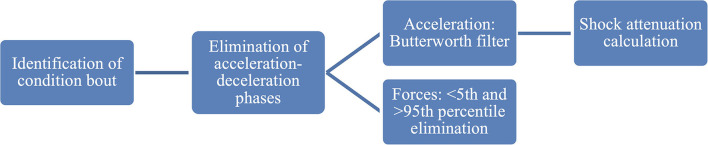
Data processing flow.

Moreover, helmet:saddle SA waves were significantly lower in both groups after the intervention at canter [F(1, 15) = 13.04, *p* < 0.001] and trot [F(1, 15) = 4.263, *p* < 0.05]. Likewise, C7:sacrum SA waves were significantly lower in both groups after the intervention at canter [F(1, 15) = 50.176, *p* < 0.001] and trot [F(1, 15) = 18.552, *p* < 0.001].

### Force Output

The two-way RM-ANOVA was conducted to examine the effects of coaching and feedback interventions on the force output at the bit and at the stirrups (Table 1). A significant increase in force output was observed in all the groups on the stirrups both at trot [F(1, 36) = 469.583, *p* < 0.001] and canter [F(1, 36) = 492.785, *p* < 0.001], but no significant changes were observed on rein forces (*p* > 0.05). No significant differences between the groups were observed (*p* > 0.05).

**Table 1:** Rein and stirrup forces (in N) before and after the coaching and feedback interventions.

			**Pre-intervention**	**Post-intervention**
Canter	Coaching	Reins	−15.53, 5.29	−14.64, 5.01
		Stirrups	−103.26, 17.19	^*^−123.98, 22.81
	Bio-feedback	Reins	−13.43, 8.72	−14.80, 5.75
		Stirrups	−93.80, 39.95	^*^−107.91, 38.32
Trot	Coaching	Reins	−7.93, 3.09	−7.15, 3.75
		Stirrups	−152.51, 14.24	^*^−154.48, 12.46
	Bio-feedback	Reins	−8.14, 5.10	−7.88, 4.33
		Stirrups	−129.90, 58.97	^*^−144.43, 49.44

* *Significant post-intervention changes (p < 0.05)*.

## Discussion

To our knowledge, this is the first study to test the effectiveness of a real-time visual kinetic feedback intervention (RKF) on the kinematics of horse riders. The study led to two major findings.

Shock attenuation (SA) was substantially improved in both groups. Moreover, there was a statistically significant improvement in SA of the C7:S1 at trot and canter, and of the helmet:saddle SA at trot in the group that received the RKF compared to the control group. The smaller SA group differences observed in the helmet:saddle region support previous findings that showed that the gross amount of shock attenuation occurs in the thoracolumbar region of the rider's spine (Elmeua González and, 2021).

Forces at the stirrups were significantly increased in both groups at trot and canter. However, higher variability was observed in the RKF group. It has previously been suggested that stirrup force is inversely correlated to total saddle force on the horse's back (Pfau et al., 2009; Peham et al., 2010); hence, higher stirrup forces can be associated with a riding technique that will be less hard on the horse's back. No significant variation on rein tension was recorded; however, the initial rein forces of the participants of the present study were substantially lower than the values seen in previous publications (Heleski et al., 2009; Egenvall et al., 2015; Eisersiö et al., 2015), leading to a smaller window of variation.

These findings suggest that introducing real-time, visual kinetic feedback can potentially reduce back spinal loading of the rider, although future research is needed. This hypothesis is supported by previous studies that have used real-time visual feedback to successfully modify gait patterns (Barrios et al., 2010; Crowell et al., 2010; van den Noort et al., 2015) and more complex tasks such as simulated rowing (Sigrist et al., 2015). This study has also highlighted the importance of the coach. Future studies looking at feedback strategies in isolation would be of great interest and would allow for a better understanding of how decisive the role of the coach is.

Whereas this study has been centered on pre-post changes following an RKF intervention, future research may benefit from retention tests with and without feedback, as it has been suggested that visual feedback that can lead to dependence and improvements may be lost if feedback is suppressed (Sigrist et al., 2013). However, it has also been proposed that concurrent visual feedback can have a positive effect on complex, sport-related tasks (Szczepan et al., 2018; Sanford et al., 2021), and that visual feedback is an effective strategy for tasks that demand a high level of task-related skills from the learner (Yamamoto et al., 2019).

## Conclusions and Practical Applications

Equestrian coaching sessions might benefit from the implementation of a real-time, visual kinetic feedback interface in the form of sports wearables. In this study, we have proposed a way which could potentially help riders of horse to optimize the ability to attenuate shockwaves that travel from the horse's back to the rider's spine; likewise, it could help achieve a stronger contact with the stirrups, but more research is needed.

## Limitations

We tried to keep uncontrolled events to a minimum and eliminate any confounding data from our recordings. Since the horse-riding biomechanics of riders have previously been shown to be strongly horse-dependent (Kuhnke et al., 2010; Eisersiö et al., 2015), we opted to use only one horse pursuing a reduction in variability; however, future assessments of independent horse-rider dyads could be an important contribution to the preliminary results.

## Data Availability Statement

The raw data supporting the conclusions of this article will be made available by the authors, without undue reservation.

## Ethics Statement

The studies involving human participants were reviewed and approved by National Ethics Committee of Republic of Slovenia. The patients/participants provided their written informed consent to participate in this study. Ethical review and approval was not required for the animal study because there were no invasive interventions for the animal and a vet check was done before and after the study. The study was not more demanding than a normal training session of the horse.

## Author Contributions

NŠ and ME conceptualized the study and the article. ME wrote the first draft. NŠ guided the process and reviewed the first edition of the manuscript. Both authors contributed to the article and approved the submitted version.

## Funding

The Slovenian Research Agency provided author NŠ with support in the form of salary through the program “Kinesiology of monostructural, polystructural, and conventional sports” [P5-0147 (B)] and the project TELASI-PREVENT [L5-1845] (Body asymmetries as a risk factor in musculoskeletal injury development: studying aetiological mechanisms and designing corrective interventions for primary and tertiary preventive care). The funders did not have any additional role in the study design, data collection and analysis, decision to publish, or preparation of the manuscript.

## Conflict of Interest

NŠ was employed by S2P, Science to Practice, Ltd. The remaining author declares that the research was conducted in the absence of any commercial or financial relationships that could be construed as a potential conflict of interest.

## Publisher's Note

All claims expressed in this article are solely those of the authors and do not necessarily represent those of their affiliated organizations, or those of the publisher, the editors and the reviewers. Any product that may be evaluated in this article, or claim that may be made by its manufacturer, is not guaranteed or endorsed by the publisher.
